# Accurate phenotyping: Reconciling approaches through Bayesian model averaging

**DOI:** 10.1371/journal.pone.0176136

**Published:** 2017-04-19

**Authors:** Carla Chia-Ming Chen, Jonathan Macgregor Keith, Kerrie Lee Mengersen

**Affiliations:** 1 Australian Institute of Marine Science, Cape Cleveland QLD, Australia; 2 ARC Centre of Excellence for Mathematical & Statistical Frontiers, Queensland University of Technology, Brisbane QLD, Australia; 3 School of Mathematical Sciences, Monash University, Clayton VIC, Australia; University of Wyoming, UNITED STATES

## Abstract

Genetic research into complex diseases is frequently hindered by a lack of clear biomarkers for phenotype ascertainment. Phenotypes for such diseases are often identified on the basis of clinically defined criteria; however such criteria may not be suitable for understanding the genetic composition of the diseases. Various statistical approaches have been proposed for phenotype definition; however our previous studies have shown that differences in phenotypes estimated using different approaches have substantial impact on subsequent analyses. Instead of obtaining results based upon a single model, we propose a new method, using Bayesian model averaging to overcome problems associated with phenotype definition. Although Bayesian model averaging has been used in other fields of research, this is the first study that uses Bayesian model averaging to reconcile phenotypes obtained using multiple models. We illustrate the new method by applying it to simulated genetic and phenotypic data for Kofendred personality disorder—an imaginary disease with several sub-types. Two separate statistical methods were used to identify clusters of individuals with distinct phenotypes: latent class analysis and grade of membership. Bayesian model averaging was then used to combine the two clusterings for the purpose of subsequent linkage analyses. We found that causative genetic loci for the disease produced higher LOD scores using model averaging than under either individual model separately. We attribute this improvement to consolidation of the cores of phenotype clusters identified using each individual method.

## Introduction

An important goal of genetic research is to understand the composition and genetic architecture of a heritable phenotype. Springboarding from the rapid reduction in the cost of genotyping and increases in computational ability, many studies have been published on the identification of different classes or subgroups of individuals based on phenotype data. In humans alone, phenotypic classes have been identified for diverse problems ranging across food acceptance [1], social behaviour (e.g. nicotine dependence) [2], psychological disorders (e.g schizophrenia) [3] and a wide variety of diseases [4–6]. The results of these phenotype analyses are often then subjected to genetic analyses in order to identify genes that are associated with, or can differentiate between, phenotype classes.

For many diseases without clear biomarkers, phenotypes are identified on the basis of clinically defined criteria. While these criteria assist in clinical diagnostics, they may not be suitable for understanding the genetic architecture of the disorder [7]. Thus different statistical methods for phenotype definition have been proposed, including latent class analysis [8], grade of membership [9], item response theory [10], factor analysis [11, 12], discriminant analysis [13] and factor mixture analysis [14]. However, different approaches can result in the identification of slightly, or sometimes substantially, different phenotype classes, and we have shown elsewhere that this can in turn significantly affect the results of subsequent analyses [15]. As phenotype identification is an indispensable precursor to most modern genetic analyses, including association studies, QTL analyses and family-based (linkage) analyses, methods for definitive phenotype calling are of fundamental importance.

**Fig 1:**
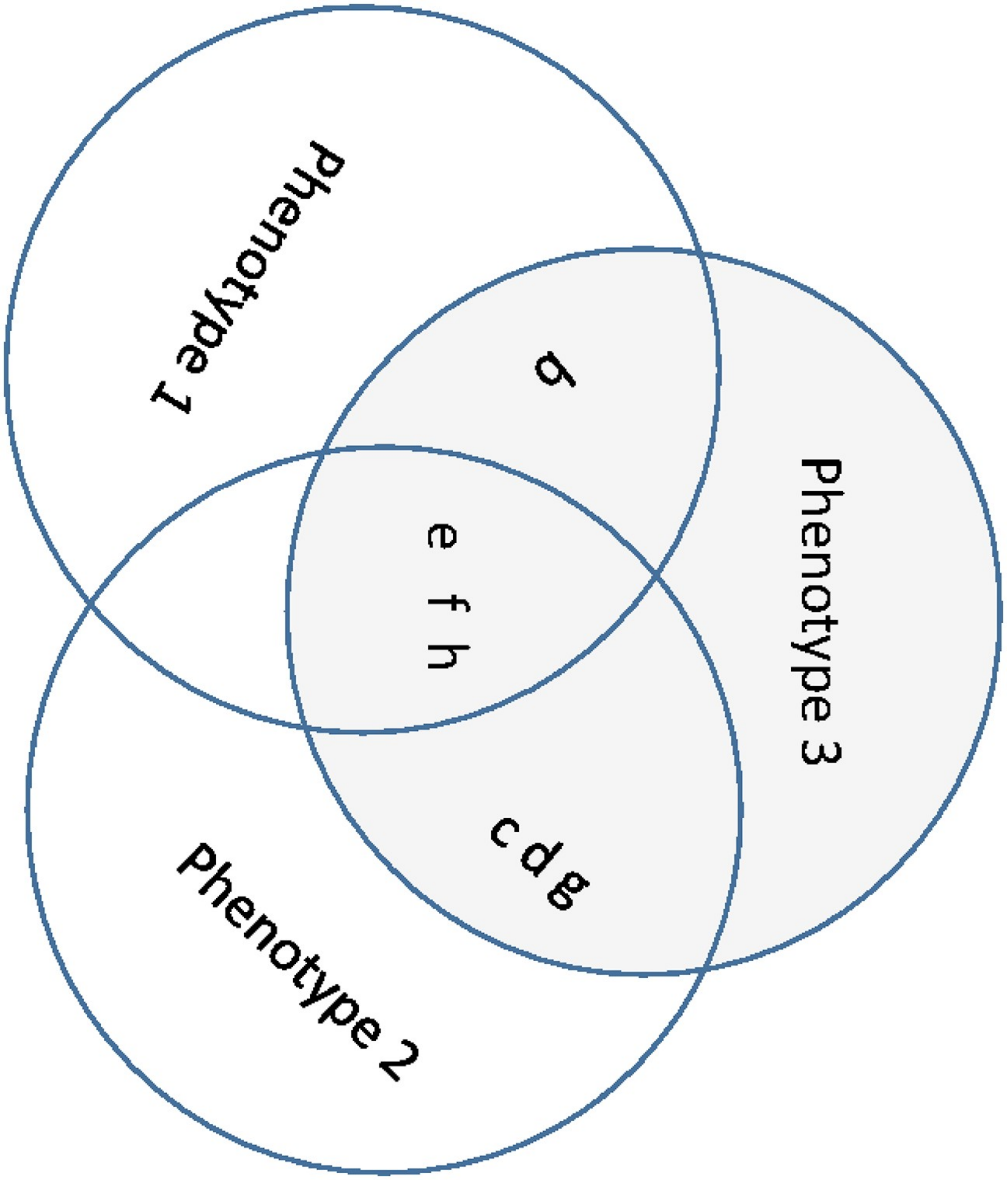
The overlapping of the traits for each of the true phenotypes. Letters b, c, d, e, f, g and h correspond to the symptoms listed in Table 4 of Greenberg [16] (also in Table 1).

To illustrate the issues arising in phenotype definition, we focus here on a simulated dataset generated for the Genetic Analysis Workshop 14 [16]. The aim of the simulation was to reflect difficulties associated with defining a phenotype for a hypothetical psychiatric condition, Kofendred Personality Disorder (KPD; see Table 4 of Greenberg et al. [16]). The disease has three different phenotypes (P1, P2, P3) and the traits characteristic of each phenotype overlap (see Fig 1). P3 has all the traits (symptoms) of P1 and P2; and P2 has nearly all the symptoms of P1. A full description of each symptom is given in Table 1.

**Table 1:** Clinical characteristics of KPD. This is the Kofendred Research Assessment Protocol for testing affected/unaffected status. Note that only symptoms b, c, d, e, f, g and h are actually associated with the disorder; the other symptoms are included to test the ability of phenotyping methods to distinguish relevant symptoms.

Indices	Description
a	Joining/founding cult
b	Fear/discomfort with strangers
c	Dislike of jokes told face to face
d	Obsession with entertainers
e	Humor impairment
f	Fascination with automobiles
g	Aversion to walking
h	Uncommunicative, contentless speech pattern
i	Fiscal irresponsibility
j	Morbid anger/fear/terror concerning rain/snow
k	Reluctance to wear clothing appropriate for subjective temperature
l	Body-image concerns/mild body dysmorphic disorder

Given the extensive overlap among the traits of the three phenotypes, it is not surprising that different statistical methods identify different clusters of individuals and symptoms. This problem can be addressed by various methods, including model selection and model averaging. In model selection, one chooses a single model, based on a criterion such as the Likelihood Ratio (LR), Akaike Information Criterion (AIC), Bayesian information criterion (BIC), Bayes Factor (BF) or posterior predictive probabilities (PPP). However, a number of researchers have recognised that this practice ignores model uncertainty [17–22]. The term *model uncertainty* refers to the unknown mathematical structure of the process generating the data; it is typically used in contrast to *parameter uncertainty*, which refers to the unknown values of the parameters of a fixed model. Ignoring model uncertainty can potentially result in underestimation of the uncertainty in the quantities of interest [23] and overemphasis on interpretation of results and association identified in the model at the expense of alternative explanations provided by closely comparable models. Furthermore, the choice of criterion for model selection can often be arbitrary and sometimes debatable; see, for example, the discussion on the validity of the Deviance Information Criterion (DIC) for different models by Spiegelhalter et al. [24].

Bayesian model averaging (BMA) potentially provides a coherent mechanism to account for model uncertainty [17, 25]. Bayesian methods quantify both model and parameter uncertainty in terms of probability. The term *posterior probability* or *posterior distribution* is used to describe the probabilities associated with parameters and models after (ie. posterior to) consideration of relevant data. The term is used in contrast to *prior probability*, which describes the probabilities associated with models before (prior to) consideration of data. The idea of BMA is to average posterior distributions estimated using different models, where the weight for each model depends on the posterior model probability. Madigan et al. [23] and Raftery et al. [26] have noted that the use of BMA can improve predictive performance. Various works have been published on methods of BMA [17, 18, 23, 26, 27]. Hoeting et al. [17] provides a thorough overview of the history, implementation, challenges and solutions for BMA. Hoeting [28] also provides a summary of BMA methodologies and lists corresponding software for carrying out the analyses.

Although the use of BMA in genetic research is not as common as in other areas of science, some published papers have incorporated these ideas in analysis. For instance, Yeung et al. [29] applied BMA for gene selection and classification of microarray data. Annest et al. [30] extended earlier research by incorporating iterative BMA for survival analysis. The use of BMA has also been implemented in the study of phylogenetics [31] and genome-wide association studies for identifying subsets of SNPs [32].

We propose here a new method to define phenotype classes. The method allows for the integration of estimated phenotypes obtained from multiple models both within and across phenotype classification approaches. Our approach to integration is similar to the “M-open perspective” discussed in Bernado and Smith [33] and Hoeting et al. [17].

The models used for illustration of the method are latent class analysis (LCA) and grade of membership (GoM). Both of these are commonly implemented in genetic research for deriving phenotypic traits of complex diseases prior to linkage or association studies, as described below. Implications for subsequent linkage analyses are assessed. Although linkage analysis has to a large extent been superseded by genome-wide association analysis, it retains some advantages over that technique, and continues to have a place in modern genetics [34].

### Data: Genetic Analysis Workshop 14

A complicated underlying genetic structure was constructed for KPD, with the involvement of four loci, denoted D1, D2, D3 and D4. Four different phenotypes are simulated: three subtypes of KPD labelled P1, P2 and P3, and an unaffected subtype. Table 2 shows the number of individuals with each phenotype (note that incidence rates of the three disease phenotypes are similar).

**Table 2:** Number of individuals with each phenotype.

Phenotype and Symptoms	Number of Individuals
P1 (b, e, f and h)	184
P2 (c, d, e, f, g and h)	193
P3 (b, c, d, e, f, g and h)	178
unaffected	853

The causal loci for each phenotype strongly overlap. The interaction of D1 and D2 results in phenotype P1; the combinations of D2 + D3 and D3 + D4 result in phenotype P2, and the combinations of D1 + D4 and D2 + D3 result in phenotype P3. The disease related loci, D1, D2, D3 and D4 are located on Chromosomes 1, 4, 5 and 9 respectively. Further details of the exact location and other genetic parameters are shown in Tables 1, 2 and 3 of Greenberg et al. [16].

Four populations were generated in the original simulation study in order to test the effect of different ascertainment schemes. Only one of the populations is included here, namely Aipotu. We included Aipotu families in our analysis when at least two of the offspring have any of the true phenotypes. There are 100 replicates and each replicate contains 100 families (approximately 700 individuals). A subset of 210 families were randomly selected from the entire simulated dataset for this demonstration.

The Genetic Analysis Workshop 14 study contained other interesting elements, such as single nucleotide polymorphism data and linkage equilibrium. However, only the microsatellite data are considered here. On average, the microsatellite markers are 7.5 centimorgan (cM) apart and there are 416 markers available without missing data.

## Materials and methods

Let Δ be a binary random variable that takes the value 1 if an individual has a particular phenotype (for example KPD phenotype P1) and takes the value 0 if not, where the individual is selected uniformly at random from a given population. Suppose clinical data can be obtained for that same individual regarding *J* symptoms. In our example *J* = 12, since we consider the 12 symptoms shown in Table 1. Let *y*_*j*_ denote a binary response to symptom *j* (*j* = 1, …, *J*) such that *y*_*j*_ = 1 indicates symptom *j* is present in the selected individual and *y*_*j*_ = 0 indicates the symptom is absent. Given a data set $Y={\left(y_{j}\right)}_{j=1}^{J}$, the model-averaged posterior distribution of Δ is given by:

$$p\left(\Delta |Y\right)=\sum_{s=1}^{S}p\left(\Delta |M_{s},Y\right)p\left(M_{s}|Y\right)$$ (1)

where *M*_*s*_ denotes one of *S* proposed models (*s* = 1, …, *S*). Using Bayes’ theorem, the probability of *M*_*s*_ given *Y* is

$$p\left(M_{s}|Y\right)=\frac{p\left(Y|M_{s}\right)p\left(M_{s}\right)}{\sum_{l}p\left(Y|M_{l}\right)p\left(M_{l}\right)}$$ (2)

where

$$p\left(Y|M_{s}\right)=\int p\left(Y|\theta_{s},M_{s}\right)p\left(\theta_{s}|M_{s}\right)d\theta_{s},$$ (3)

which is called the marginal likelihood for model *M*_*s*_. Here *θ*_*s*_ denotes the model parameters of model *s*. In the context of this paper, as described in the section on Models and Settings below, *S* = 2, *M*_1_ is the LCA model and *M*_2_ is the GoM model.

In the model-averaging method proposed here, we assume that one can generate an estimate *ϕ*_*is*_ of *p*(Δ = 1|*M*_*s*_, *Y*_*i*_) for each model *M*_*s*_ and for an individual *i* with symptoms indicated by *Y*_*i*_. This is the probability that individual *i* has the phenotype of interest, here KPD phenotype P1, given the symptoms and model *M*_*s*_. In practice, we generate a sequence of estimates $\phi_{is}^{t}$ of this probability at each iteration *t* of a Markov chain Monte Carlo (MCMC) technique, and then define *ϕ*_*is*_ to be the unweighted average of $\phi_{is}^{t}$ over post-burn-in iterations.

Eq (1) then generates a ‘model-averaged’ estimate *ϕ*_*i*_ of *p*(Δ = 1|*Y*):

$$\phi_{i}=\sum_{s=1}^{S}\phi_{is}p\left(M_{s}|Y\right)$$ (4)

The ‘weights’ on the right-hand side are estimated using Eqs (2) and (3). This value can be used as the phenotype of individual *i* in subsequent linkage analysis. In our past experience with migraine data [15], we observed that it makes no difference whether the phenotype used in linkage analysis is a binary variable indicating the status of a patient (affected/not affected) or a continuous variable representing the probability of an individual having the disease or disorder, considering all symptoms. Here it will be convenient to use the latter representation.

In practice, it may be difficult to evaluate the marginal likelihood of a model *M*_*s*_, because the integral in Eq (3) is intractable. Various methods have therefore been proposed for approximating the marginal likelihood [18, 35–37]. Here we use the Laplace-Gibbs approximation [38], a variant of the Laplace-Metropolis algorithm.

The Laplace-Metropolis algorithm is based on Laplace’s asymptotic approximation

$$\int e^{\log(p\left(Y|\theta ,M_{s}\right)p\left(\theta |M_{s}\right))}d\theta \approx {\left(2\pi \right)}^{\frac{d}{2}}{|H^{*}|}^{\frac{1}{2}}p\left(Y|\theta^{*},M_{s}\right)p\left(\theta^{*}|M_{s}\right)$$ (5)

where *d* is the dimension of the parameter vector *θ*, θ* is the posterior mean value of *θ* and H* is the minus inverse of the Hessian matrix, which is evaluated at θ*. Due to the difficulties in analytical estimation of θ* and H*, Raftery [39] suggests the use of the posterior simulation outputs to estimate the quantities required for Eq (5), and called it a Laplace-Metropolis algorithm. The Laplace-Gibbs approximation is similar, but estimates are derived from Gibbs rather than Metropolis-Hastings samples. Lewis and Raftery [38] provide four methods for estimating θ*, which are simple to implement.

### Models and settings

We chose two statistical models, namely latent class analysis and grade of membership, to demonstrate the model-averaging method proposed in the previous section. These two models are commonly used for deriving phenotypes of complex diseases. Both are mixture models and likelihood-based approaches. In this study, both models are considered in a Bayesian framework.

For LCA, following McCutcheon [8], suppose that there are *n* individuals and *J* symptoms (*i* = 1, …, *n*; *j* = 1, …, *J*). Let *y*_*ij*_ denote a binary response of individual *i* to symptom *j*, such that *y*_*ij*_ = 1 indicates symptom *j* is present in person *i*. Let *K* denote the number of symptom clusters, that is, the number of distinct phenotypes. Then LCA is a mixture of Bernoulli distributions,

$$p\left(Y_{i}|\lambda ,p\right)=\sum_{k=1}^{K}p_{k}f\left(Y_{i}|\theta \right)=\sum_{k=1}^{K}p_{k}\prod_{j}^{J}{\left(\lambda_{kj}\right)}^{y_{ij}}{\left(1-\lambda_{kj}\right)}^{1-y_{ij}}$$ (6)

where *p*_*k*_ is the proportion of individuals in the population with phenotype *k*, *Y*_*i*_ = {*y*_*i*1_, *y*_*i*2_, …, *y*_*ik*_} is a vector of symptom indicators for individual *i* and *λ*_*kj*_ is the probability of a positive response on symptom *j* for a subject with phenotype *k*. Non-informative priors were adopted, namely

$${\left(p_{k}\right)}_{k=1}^{K}∼Dirichlet{\left(1,...1\right)}_{k};\;\lambda_{kj}∼Beta\left(1,1\right).$$ (7)

(The term *non-informative prior* is used to describe a prior distribution that represents a state of minimal knowledge about a parameter, here the values *p*_*k*_ and *λ*_*kj*_.)

It is convenient to define indicator variables *z*_*ik*_ which take the value 1 if individual *i* has phenotype *k*, and 0 otherwise. The advantage of this is that the conditional posterior distributions of *p* and *λ* take a simple form—they are Dirichlet and Beta distributions.

$$\begin{array}{cc} p_{k} & ∼Dirichlet(\sum_{i}z_{i1}+1,..\sum_{i}z_{iK}+1)\\ \lambda_{kj} & ∼Beta(\sum_{i}\left(z_{ik}y_{ij}\right)+1,\sum_{i}\left(z_{ik}-z_{ik}y_{ij}\right)+1) \end{array}$$ (8)

where

$$z_{ik}∼multinomial\left(\delta_{i1},...\delta_{iK}\right);\;\delta_{ik}=\frac{p_{k}f\left(Y_{i}|\theta \right)}{\sum_{k}p_{k}f\left(Y_{i}|\theta \right)}$$

More detail regarding the derivation of these conditional posterior distributions is provided as supplementary material.

These conditional distributions can then be used as the basis of an MCMC approach [40], specifically a Gibbs sampler, to generate estimates of *p*_*k*_ and *λ*_*kj*_. Note that the term *δ*_*ik*_ defined above is the posterior probability that individual *i* has phenotype *k*, and thus provides the estimate *ϕ*_*i*1_ of *P*(Δ = 1|*M*_1_, *Y*_*i*_) required for model averaging, where Δ is an indicator variable for phenotype *k*, and *M*_1_ is LCA.

For GoM, following Erosheva [41], let *g*_*ik*_ be a latent variable of membership score, representing the probability that individual *i* belongs to cluster *k*. This immediately provides the estimate *ϕ*_*i*2_ of *P*(Δ = 1|*M*_2_, *Y*_*i*_), where *M*_2_ is GoM. Constraining the number of levels of responses in symptom *j* to 2, GoM is similar to a mixture of Bernoulli distributions,

$$Pr\left(Y_{i}|\gamma ,g\right)=\prod_{j=1}^{J}\{{\left(\sum_{k}g_{ik}\gamma_{kj}\right)}^{y_{ij}}{\left(1-\sum_{k}g_{ik}\gamma_{kj}\right)}^{\left(1-y_{ij}\right)}\}$$ (9)

where *γ*_*kj*_ is similar to *λ*_*kj*_ of the LCA model, and is the probability of having symptom *j* for an individual in cluster *k*. Again, non-informative priors are used here,

$$g_{ik}∼Dirichlet{\left(1,...1\right)}_{k};\;\gamma_{kj}∼Beta\left(1,1\right)$$ (10)

We introduce a vector of *J* categorical variables *ω*_*i*_ = (*ω*_*i*1_, …, *ω*_*iJ*_) for each individual *i*. Each *ω*_*ij*_ can take on *K* values from {1, …, *K*}. The latent class is then defined as *ω*_*i*_ ∈ *Ω* = {1, 2, …, *K*}^*J*^. It is also convenient to define *ω*_*ijk*_ = 1 if *ω*_*ij*_ = *k* and *ω*_*ijk*_ = 0 otherwise.

A Gibbs sampler is again used to estimate the model parameters based on the conditional posterior distributions,

$$\begin{array}{cc} g_{ik} & ∼Dirichlet(\sum_{j}\omega_{ij1}+1,\ldots ,\sum_{j}\omega_{ijK}+1)\\ \gamma_{kj} & ∼Beta(\sum_{i}\left(\omega_{ijk}y_{ij}\right)+1,\sum_{i}\left(\omega_{ijk}-\omega_{ijk}y_{ij}\right)+1) \end{array}$$ (11)

where

$$\omega_{ijk}∼multinomial\left(\kappa_{ij1},\ldots ,\kappa_{ijK}\right);\;\kappa_{ik}\propto g_{ik}\gamma_{kj}^{x_{ij}}{\left(1-\gamma_{kj}\right)}^{1-x_{ij}}$$

The model averaging method we describe in this paper is in principle able to cope with a large number of clusters, (i.e. large *K*). However, this can be computationally burdensome. Therefore we suggest limiting *K* to small values. In the simulated dataset, the known true number of clusters is four (three subtypes of KPD and an unaffected subtype)

We used R **poLCA** and **sirt** packages to fit LCA and GoM to the simulated dataset, and found BIC was lowest when *K* = 3 in both models (Table 3). Note BIC is a common criterion for comparing models, with lower scoring models typically preferred. For demonstration purposes, the number of clusters is limited to three for both models in what follows.

**Table 3:** Bayesian information criteria for LCA and GoM with number of components varying from 2 to 6.

Number of components	LCA	GoM
2	13711.18	13735.82
3	12176.3	12275.69
4	12187.76	12428.85
5	12205.95	12688.61
6	12320.34	13180.32

One challenge of averaging over clustering models is the comparability of clusters between models. In our experience, when *K* = 2, most clustering methods tend to identify groups with extreme characteristics, that is, one group of individuals with all or most symptoms and a second group with limited or no symptoms. Consequently, clusters are typically comparable between models. However, when *K* ≥ 3, clusters are potentially not comparable between models.

One way to assess cluster comparability is to use a similarity matrix. Various ensemble methods are proposed in the clustering literature [42–45] with the focus on the similarity between observations. In our study, the main interest is to estimate the probability of each individual belonging to a specific cluster; it is therefore more important to compare the similarity between clusters than the similarity between observations. When it is clear that a cluster identified using one model is not comparable to any of the clusters identified by another model, we recommend that such clusters not be merged, but remain distinct for the purpose of model averaging. We demonstrate below how this can be done.

As noted above, when *K* = 2 most clustering methods tend to identify one cluster representing affected individuals and one representing those not affected; therefore it is natural to see the probability of belonging to the affected cluster as the phenotypic trait. However, the definition of phenotypic trait is less straightforward when *K* ≥ 3, which may indicate there are subtypes of the disease/disorder. Therefore, when *K* ≥ 3, we propose defining up to *K* phenotypic traits; corresponding to the probability of belonging to each of the affected clusters (subtypes). Note this assumes that one or more of the clusters can be identified as representing individuals that are clearly not affected.

Given no information to support an alternative decision, we gave equal prior probability to each model. It is known that H* is asymptotically equal to the posterior covariance matrix when sample size tends to infinity [46], so we approximated H* by the estimated covariance matrix of the posterior simulation.

Given the familial pedigree and microsatellite data in the case study, QTL linkage was used to identify important markers [47]. This identifies the linkage between the markers and disease loci by regressing the squared trait differences of sib-pairs on identity-by-descent allele-sharing. A sib-pair that shares more alleles is expected to show a similar phenotype, that is, a smaller difference in trait value. A separate linkage analysis was performed for each of the affected clusters. Note that there is no ‘correct’ choice of cluster: each affected cluster may represent a distinct sub-type and may be associated with distinct loci, though sub-types may also have loci in common. The linkage analysis was carried out using MERLIN-qtl [48].

The algorithms were implemented using the C++ programming language. Three MCMC chains were generated for each model with 500,000 iterations. The first 490,000 iterations were treated as burn-in samples and were removed from analysis. Only 10,000 samples were retained because the large number of parameters in the models, especially GoM, made storage of samples expensive in terms of memory requirements. It was not necessary to discard 490000 samples as burn-in, as convergence was apparent from time series plots of the deviance well within the first half of the run. This very long burn-in does, however, provide strong confidence that convergence has occurred. An alternative would have been to draw 10,000 sub-samples uniformly at random or evenly spaced from the second half of the chain, a practice known as ‘thinning’.

## Results

**Fig 2:**
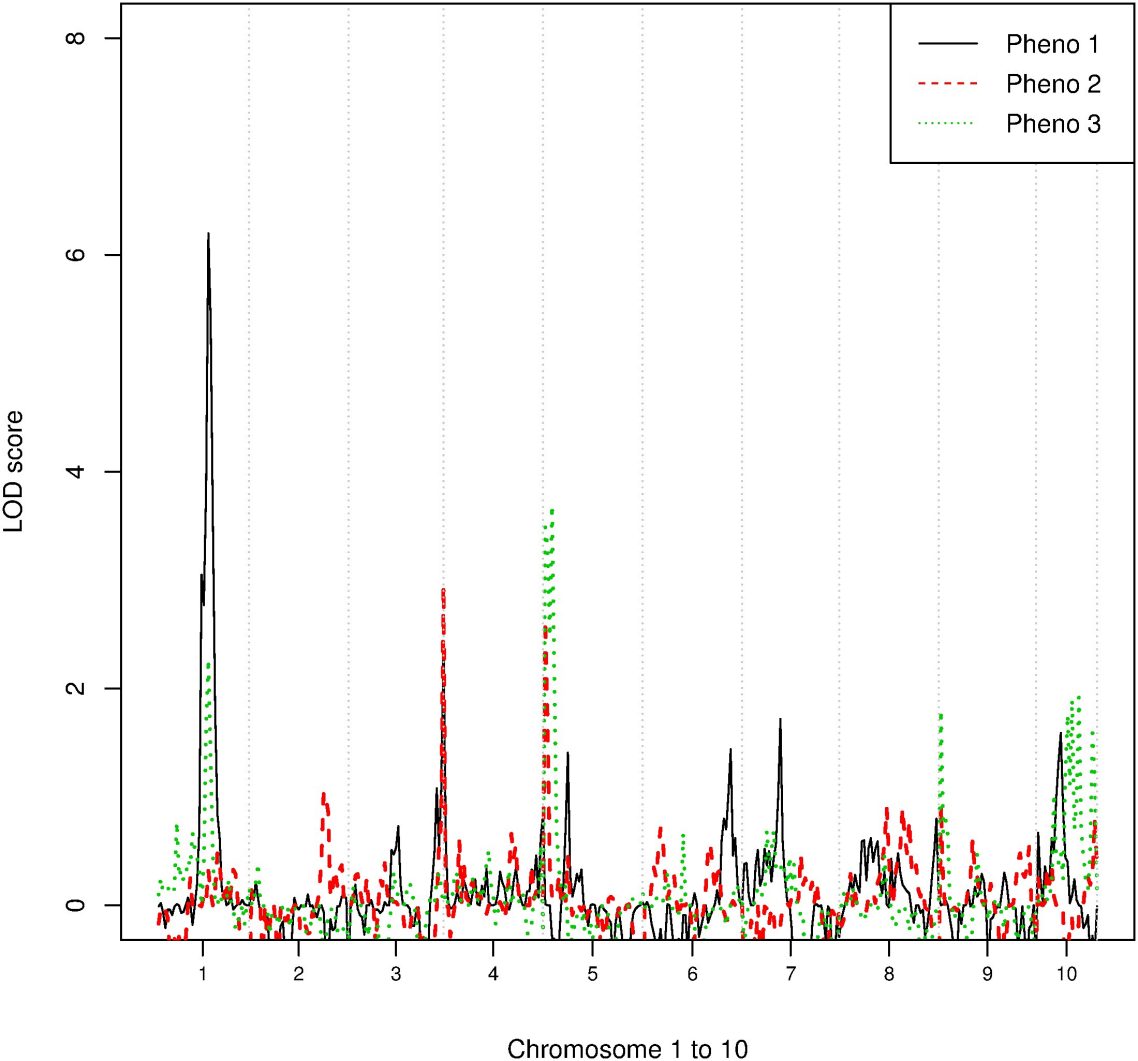
LOD scores of the phenotypes for each of the microsatellite markers across ten chromosomes. P1, P2 and P3 indicate Phenotype 1, 2 and 3. The dotted line is the LOD score of Phenotype 1 estimated using MERLIN-qtl; the dashed-line is the LOD score of Phenotype 2 and the solid line is the LOD score of Phenotype 3. This is used as a benchmark for comparing the results of proposed methods.

Considering that the KPD data was simulated with epistasis effects, and given that when testing for interactions QTL linkage analysis is usually limited to detecting deviance for an additive model at a single locus (dominance) rather than testing for dependence between loci (epistasis), it is important to first evaluate the capability of MERLIN to identify the actual loci when the true phenotypes of individuals are known. Fig 2 shows the LOD scores of phenotypes for each of the microsatellite markers across ten chromosomes. The solid, dashed and dotted lines represent the LOD scores of Phenotypes 1, 2 and 3, respectively. MERLIN was able to clearly identify the two correct disease loci of Phenotype 1, with LOD scores above 2.4. For P3, MERLIN was able to identify two of the three major loci. It was also able to identify two of the three disease loci for P2. One possible explanation for failing to detect some loci is that individual genotypes were simulated based on each symptom instead of phenotype classes, therefore when only a small sample is included in the study (one tenth of all simulations) conducting linkage analysis on a binary scheme is unable to detect some important loci.

**Fig 3:**
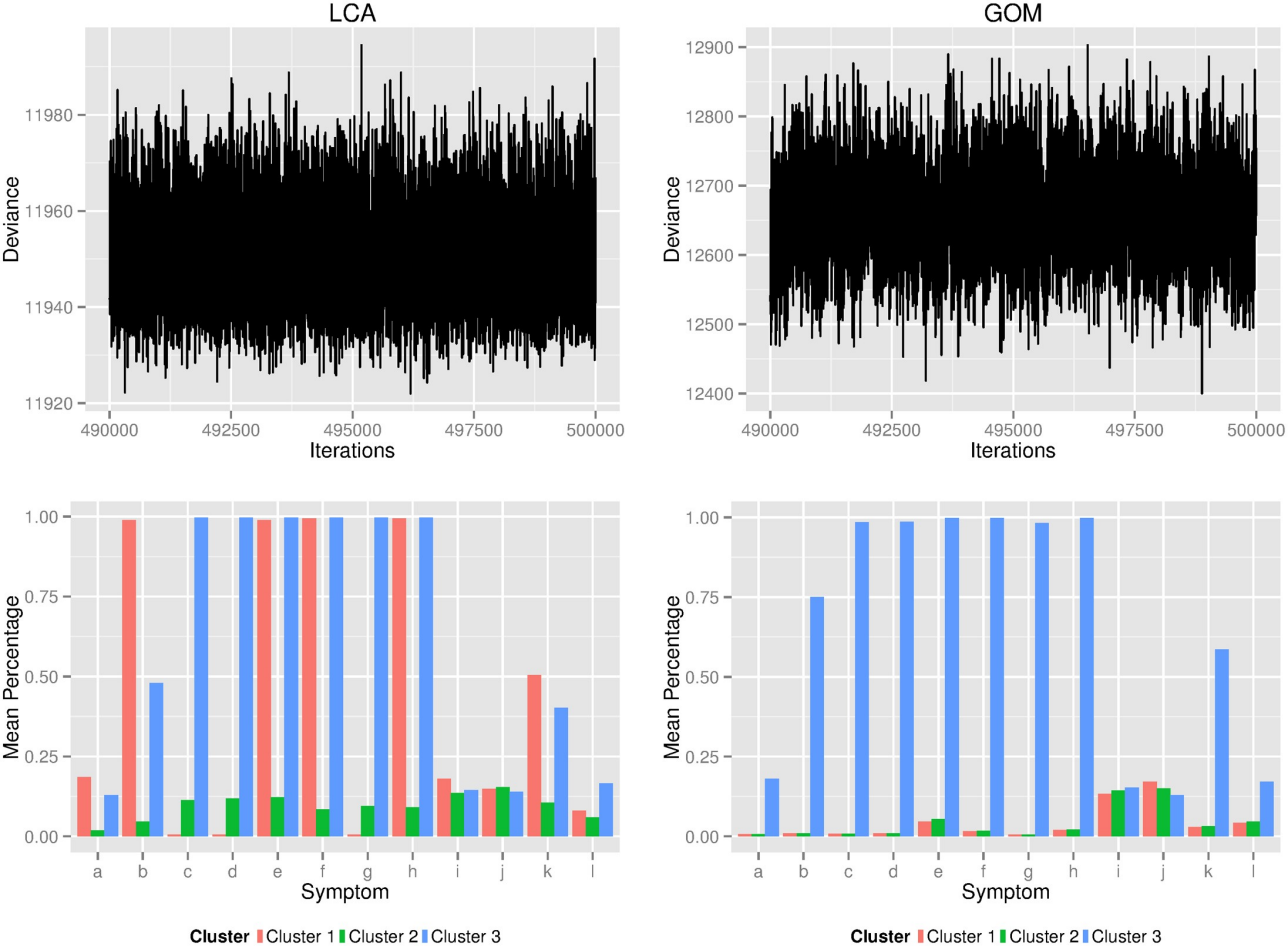
The characteristics of clusters derived from different statistical models. Plots on the left are deviance and posterior means of symptom prevalence in clusters of LCA and plots on the right are deviance and symptom prevalence in clusters of GoM.

Using the 210-families KPD data set and a Gibbs sampler, MCMC chains converged for both the LCA and GoM models within 500,000 iterations (top of Fig 3) and marginal distributions were estimated using the last 10,000 iterations. At the bottom of Fig 3 are posterior mean values of *λ*_*jk*_ for LCA and posterior mean values of *γ*_*jk*_ for GoM. Estimates of the phenotypes *ϕ*_*is*_ under LCA, GoM and the combined phenotypes are available at https://github.com/cewels/PlosOne_BMA_paper.

These figures illustrate the ability of both LCA and GoM to identify true phenotype classes. Comparing these results with symptom combinations of true phenotypes (Fig 1), the clusters identified by LCA are more aligned with true phenotypes than those identified by GoM. Clusters 3 and 1 found by LCA correspond to true phenotypes 3 and 1 respectively, and cluster 2 corresponds to the non-KPD cluster. In contrast, GoM is only effective in separating the extreme classes. Cluster 3 of GoM is equivalent to true phenotype 3, however clusters 1 and 2 of GoM both correspond to the non-KPD cluster and have nearly identical characteristics. It is unlikely that this can be attributed to lack of convergence considering the results remained unchanged when the number of MCMC iterations was doubled. It might be asked whether there is any advantage in model averaging when the phenotypes obtained using LCA are so close to the true phenotypes. However, we know this only because we are using simulated data; it is not known whether LCA always gives more accurate phenotypes than GoM, or whether LCA always gives results that so closely approximate true phenotypes.

In general, if one method produces superior phenotypes to the other, then the model-averaged phenotypes will be intermediate. But which method is superior will not be known, and model-averaged phenotypes thus reflect model uncertainty. Nevertheless, we show below that model-averaged phenotypes can sometimes result in higher LOD scores than can be obtained using either LCA or GoM separately.

For each phenotype, the sensitivities and specificities of LCA and GoM can also be estimated using this simulated data. Both models produce phenotypes in the interval (0, 1), specifically $\phi_{i}^{\left(1\right)}$, $\phi_{i}^{\left(2\right)}$ and $\phi_{i}^{\left(3\right)}$, representing probabilities of belonging to subgroups 1, 2 and 3. We used a threshold of 0.5 and when $\phi_{i}^{\left(k\right)}\geq 0.5$, we assigned individual *i* to phenotype *k*. Table 4 shows the sensitivities and specificities of LCA, GoM and their combination for phenotypes 1 and 3. As neither LCA nor GoM can identify phenotype 2, sensitivity and specificity for this phenotype are not available. All models achieved perfect sensitivity and reasonable specificity for phenotype 3, with GoM having the lowest specificity of 0.75. (However, in fairness it should be noted that the specificity of GoM was the same as for LCA for the combined method when a threshold value of 0.7 was used.) Note that LCA achieves near perfect sensitivity and specificity for both phenotypes, but again it should be stressed that we can know this only because the data is simulated. In practice, this would not be known; hence the need for model averaging to account for model uncertainty. The sensitivity and specificity obtained with the combined models was identical to those obtained for LCA only due to the choice of threshold value and weight distributions.

**Table 4:** Sensitivities and specificities of the LCA, GoM and combined method for Phenotype 1 and Phenotype 3 of LCA. None of the models identified a class with structure similar to phenotype 2.

Phenotype	Model	Sensitivity (%)	Specificity (%)
3	LCA	1.0	0.8431
	GoM	1.0	0.7504
	Combined	1.0	0.8431
1	LCA or Combined	1.0	0.9984

Using Eqs (2) and (3) with Laplace-Gibbs to estimate the marginal likelihood of each model, we obtained model weights of 0.82 for LCA and 0.18 for GoM. This confirms our expectation that LCA is the more appropriate model for this data set, and is consistent with the lower BIC value of this model. However, it also demonstrates that the GoM model still contributes to the phenotype value.

Clusters 1 and 2 of GoM and Cluster 2 of LCA have the lowest symptom prevalences; these are excluded in the following linkage analysis. According to Fig 3, the symptoms characteristic of cluster 3 of LCA and cluster 3 of GoM are nearly identical, therefore these two clusters were averaged prior to subsequent analyses (we label this model averaged cluster K2). Note that cluster 3 of LCA and cluster 3 of GoM correspond to high incidence of symptoms c, d, e, f, g, h and to some extent b and k. Cluster K2 therefore corresponds to true phenotype 3, which involves all of these symptoms except k (Fig 1). Cluster 1 of LCA does not correspond to any of the GoM clusters, therefore we retained this cluster as distinct (we re-labelled this cluster K1). According to Fig 3, the symptoms characteristic of this cluster are b, e, f, h and to some degree k. Apart from k, these are precisely the symptoms of true phenotype 1 (Fig 1). We then carried out two independent linkage analyses to identify loci associated with each of *K*1 and *K*2.

**Fig 4:**
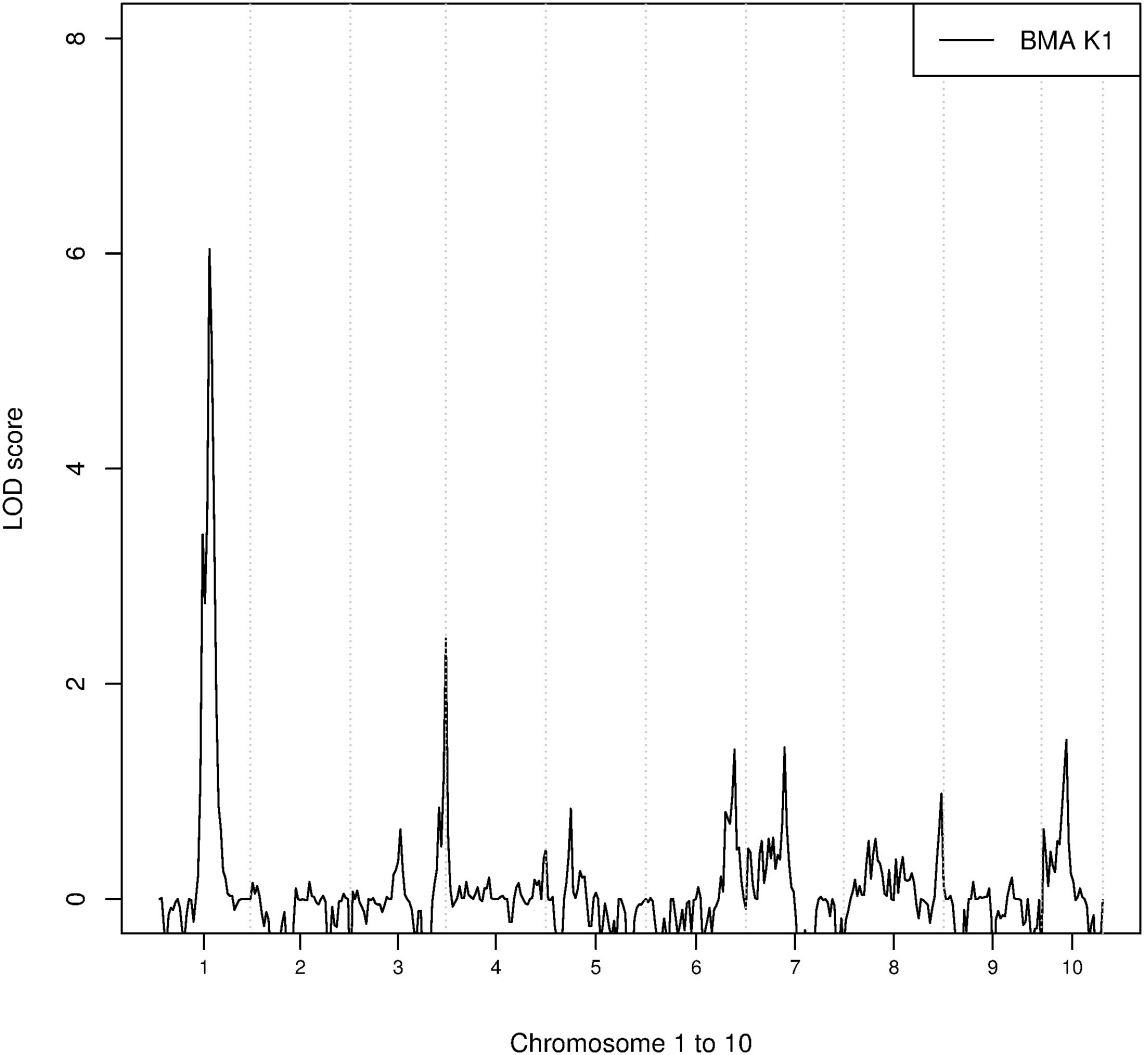
LOD scores at each satellite marker for phenotype K1.

Fig 4 shows the LOD score of quantitative trait linkage analysis for cluster K1, which is nearly identical to the LOD scores of true phenotype 1 (Fig 2). This is not surprising given the high sensitivity and specificity of K1 relative to true phenotype 1. Hence the results of the subsequent analysis identified the same disease loci: markers in the region from D01S0020 to D01S0025 (with the highest LOD score of 6.35 at D01S0023) and a second disease locus around marker D03S0127.

**Fig 5:**
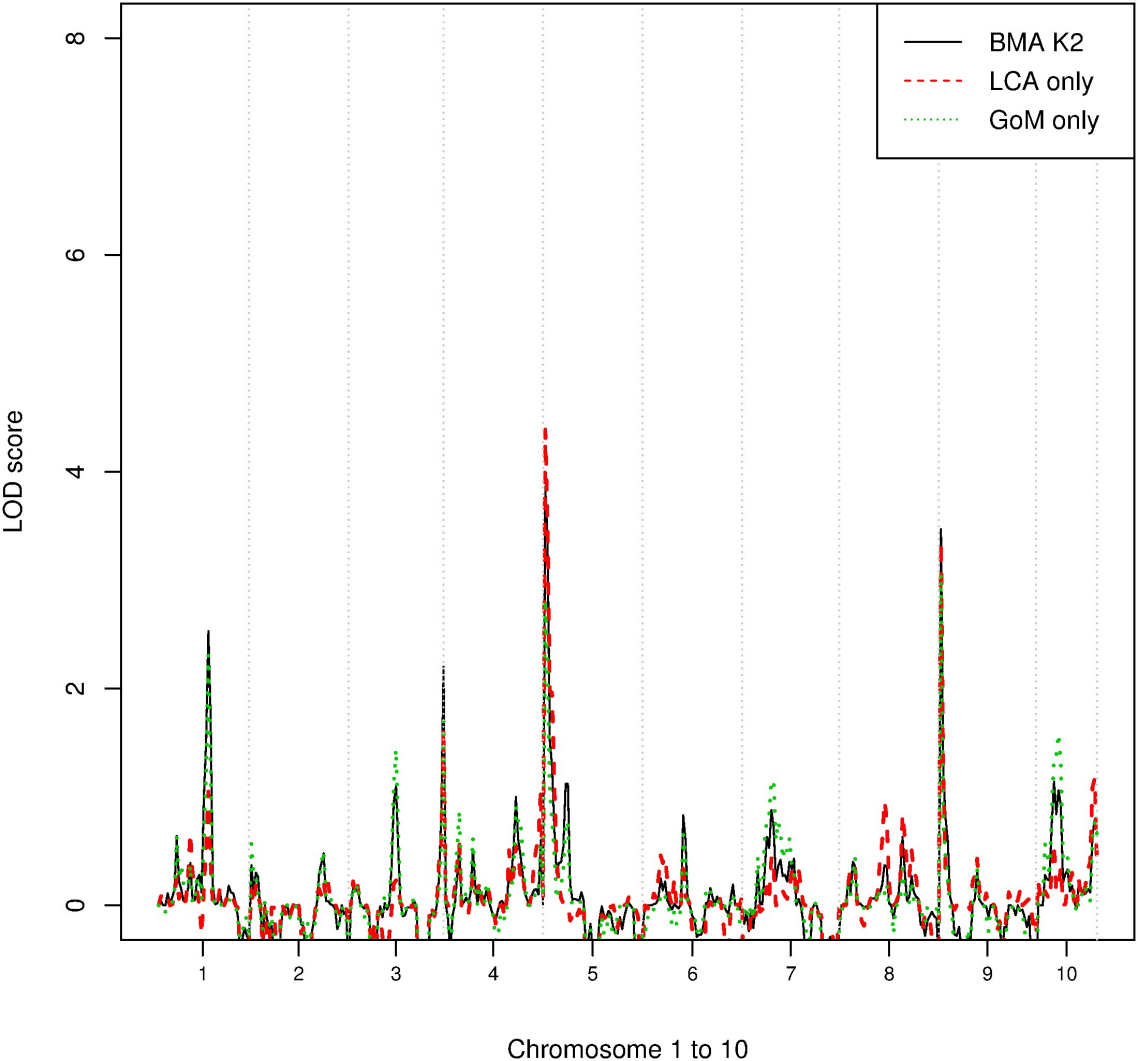
LOD scores at each satellite marker for phenotypes estimated after model averaging. The black solid line shows the LOD scores obtained for K2 estimated using model averaging, the red dashed line shows the LOD scores of cluster 3 of LCA and the green dotted line is the LOD score using phenotype derived from GoM alone (cluster 3 of GoM).

**Fig 6:**
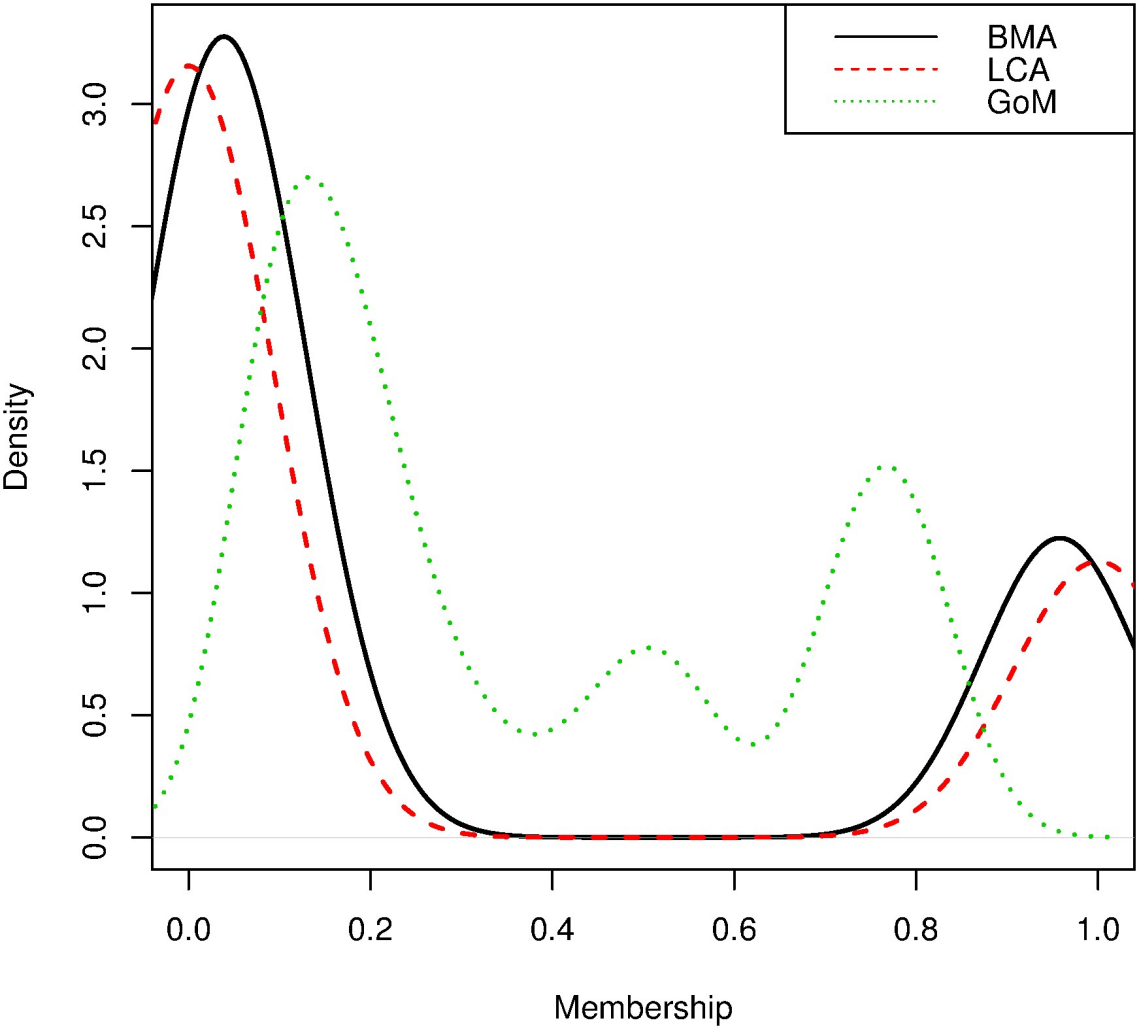
Density of the estimated phenotypes K2. The black solid line represents the distribution (over individuals) of the averaged phenotype weighted according to Laplace-Gibbs; dashed and dotted lines represent the distributions of the posterior mean of the phenotype predicted by LCA and GoM.

Unlike K1, phenotype K2 is derived from averaging $\phi_{i}^{\left(3\right)}$ over two independent models with heavier weight placed on LCA. Linkage analysis of K2 successfully identified all four disease loci (Fig 5) with LOD scores all above 2.0, compared to only three marker regions identified using the phenotype estimated using LCA alone. That the model averaged cluster identified more of the relevant loci can be attributed to the fact that LCA must definitely assign subjects to classes whereas GoM allows fuzzy memberships (Fig 6). Incorrect assignments made using LCA alone reduce the power of the subsequent genetic analysis. The linkage results obtained using the K2 phenotype also identified disease loci that were not identified when the phenotype was derived using the actual criteria (i.e Chromosome 3, Fig 2).

When using the phenotype derived from GoM alone, the number of marker regions associated with the disease tends to be overestimated. For instance, markers D10S0399 and D03S0106 are not true disease markers but the LOD score for these two loci are around 1.5. The LOD scores obtained for some of the true KPD-associated loci using the combined phenotypes were actually higher than the LOD scores obtained using LCA or GoM derived phenotypes. This may seem strange, as one might suppose that the LOD scores obtained using the model-averaged phenotypes must lie between those obtained using each of the models separately. However, it is not the LOD scores, but the phenotypes, for which model averaging is performed. What has happened is that the separation between affected and unaffected individuals has been improved by model averaging: cores of clusters corresponding to individuals identified as affected or unaffected by both LCA and GoM have been consolidated, whereas the periphery of clusters corresponding to individuals whose affectedness status differed according to LCA and GoM have been shifted to moderate phenotype values.

## Discussion

The study of diseases with complex etiology demands a clear, statistically relevant definition of the phenotype prior to genetic analysis. Here we proposed a multi-model approach and provided an algorithm for integrating phenotypes using Bayesian model averaging as a foundation. In the examples, we selected two models which have in common a latent class framework, but are very different in terms of parameter spaces and identification of class membership (probability of belonging to phenotype clusters).

An advantage of model averaging is the consolidation of the cores of the clusters commonly identified under the different models and clearer reflection of the model uncertainty at the boundaries of the clusters. Consequently, in the subsequent linkage analysis, loci which are strongly differentiated at the cluster cores may have stronger LOD scores under the combined model than under any individual model. Although other methods to consolidate clusters in such a manner may be possible, it is clear that model selection, the main alternative to model averaging, does not achieve this desirable effect.

Of course, other approaches to combining the results of phenotype and linkage analyses may be considered. An example is running the linkage analyses for each of the separate phenotype models and combining the linkage results. This would result in a simple weighted averaging of the LOD scores at each locus. Under this method, however, the LOD score of each locus will necessarily lie within the range of the LOD scores obtained under the individual models. While this may be appealing in one sense, it can be argued that the combination of methods should allow for increased inferential capability. As demonstrated in our example, it is possible by model averaging prior to linkage analysis to obtain LOD scores for the combined phenotype that are higher than would be obtained with either model used separately.

In our example, the number of clusters selected for each model was determined prior to the phenotype and linkage analyses. Although the actual number of clusters is four (three subtypes of KPD and an unaffected subtype), subtle distinctions between subtypes are difficult to detect. Although the results are not shown here, we analysed the simulated KPD data with *K* = 4 using the LCA model. Again three of the true clusters were identified (P1, P3, unaffected) but P2 did not correspond to the remaining cluster. It is also interesting to note that even when the true clusters are identifiable, the linkage analysis may not always identify the important genes for each subtype (Fig 2). This is due to the complex genetic framework implemented in this data simulation. The linkage analysis implemented here has limited capability to identify “modifying” loci, which switch between phenotypes 1 and 2. This affects the penetrance of phenotype 2.

One challenge of implementing model averaging methods for three or more clusters is the comparability of clusters found by different models. In this study, we propose to overcome this challenge by first identifying the characteristics of clusters and then merging membership of the clusters between models, if and only if the characteristics of clusters are highly compatible (e.g Cluster 3 of LCA and GoM). This implies characteristics of the clusters remain little changed after averaging. Similar approaches are proposed in other studies. Russell et al. [45] propose constructing a similarity matrix based on the probabilities that any pair of observations belong to the same cluster when averaging mixtures of Gaussian distributions; while Wei et al. [44] proposed the use of adjusted Rand index to merge components based on a reference model.

Further research is also warranted into the impact of different model evaluation strategies when the models are strongly contrasting with respect to number of parameters. Other approaches may be more applicable, and other approximations to the marginal likelihoods [18, 49–51] may be investigated. The methods proposed in this paper may be more applicable when the number of parameters in the two models are more comparable, for example, item response theory [52] and GoM or mixture models with different distributions.

There are other open questions about the methods proposed in this paper, such as the choice of priors. The Bayes factor has been shown to be sensitive to the choice of priors [18]; thus it is important to validate the prior distribution with sensitivity analysis. Moreover, in the examples of this paper, the subsequent analysis is restricted to genome-wide linkage analysis implemented in MERLIN-qtl. The linkage analysis by Haseman and Elston [47] assumed that the markers are independent, so lacks ability to detect an interaction effect. Although linkage analysis shows great success in mapping the genes for Mendelian disorders, to detect the finer resolution of the putative risk susceptibility loci through linkage analysis is only feasible with the availability of large recombination events from large pedigrees. Therefore, the feasibility of detecting variants with low penetrance using linkage methods is questionable [53]. Furthermore, the methods may also be suitable for genetic association studies and other methods that rely on accurate phenotype calling.

Another possibility for further research is to perform simultaneous linkage analysis and phenotype calling. An important advantage of this approach is that it would enable the genotype data to influence the phenotype classification, potentially enabling a clearer separation between phenotype groups than would be possible using phenotype data alone. However, linkage analysis is only one type of genetic analysis that requires accurate phenotype calling, and there may be an advantage in identifying phenotypes that are generally applicable, rather than tailored to a specific genetic analysis technique.

## Supporting information

S1 File Derivation of conditional distributions. (PDF)

## References

[1] Drewnowski A and Rock CL. The influence of genetic taste markers on food acceptance. Am J Clin Nutr 1995;62, 506–511 7661111 10.1093/ajcn/62.3.506

[2] Bierut LJ, Madden PAF, Breslau N, Johnson EO, Hatsukami D, Pomerleau, et al Novel genes identified in a high-density genome wide association study for nicotine dependence. Hum Mol Genet 2007;16, 24–35. 10.1093/hmg/ddl441 17158188 PMC2278047

[3] Hallmayer JF, Jablensky A, Michie P, Woodbury M, Salmon B, Combrinck J, et al Linkage analysis of candidate regions using a composite neurocognitive phenotype correlated with schizophrenia. Mol Psychiatr 2003;8, 511–523. 10.1038/sj.mp.4001273 12808431

[4] Nyholt DR, Gillespie NG, Heath AC, Merikangas KR, Duffy DL, and Martin NG. Latent class and genetic analysis does not support migraine with aura and migraine without aura as separate entities. Genet. Epidemiol. 2004;26, 231–244. 10.1002/gepi.10311 15022209

[5] Corder EH and Woodbury MA. Genetic heterogeneity in Alzheimer’s disease: A grade of membership analysis. Genet Epidemiol 1993;10, 495–499. 10.1002/gepi.1370100628 8314050

[6] Imperatore G, Hanson RL, Pettitt DJ, Kobes S, Bennett PH, and Knowler WC. Sib-pair linkage analysis for susceptibility genes for microvascular complications among Pima Indians with type 2 diabetes. Pima diabetes genes group. Diabetes 1998;47, 821–830. 9588456 10.2337/diabetes.47.5.821

[7] Wessman M, Terwindt GM, Kaunisto MA, Palotie A, and Ophoff RA. Migraine: a complex genetic disorder. The Lancet Neurol. 2007;6, 521–532. 10.1016/S1474-4422(07)70126-6 17509487

[8] McCutcheon AL. Latent Class Analysis Quantitative Applications in the Social Science. (Newbury Park: Sage Publications); 1987.

[9] Manton KG, Woodbury MA, and Tolley HD. Statistical applications using fuzzy sets. (Wiley); 1994.

[10] Eaves L, Silberg J, Foley D, Bulik C, Maes H, Erkanli A, et al Genetic and environmental influences on the relative timing of pubertal change. Twin Res 2004;7, 471–481. 10.1375/1369052042335278 15527663

[11] Cho MH, Washko GR, Hoffmann TJ, Criner GJ, Hoffman EA, Martinez FJ, et al Cluster analysis in severe emphysema subjects using phenotype and genotype data: an exploratory investigation. Respir Res. 2010;11, 30 10.1186/1465-9921-11-30 20233420 PMC2850331

[12] Roy K, Smith J, Kolsum U, Borrill Z, Vestbo J, and Singh D. COPD phenotype description using principal components analysis. Respir Res 2009;10, 41 10.1186/1465-9921-10-41 19480658 PMC2698901

[13] Zinn AR, Roeltgen D, Stefanatos G, Ramos P, Elder FF, Kushner H, et al A Turner syndrome neurocognitive phenotype maps to Xp22.3. Behav Brain Funct 2007;3, 24 10.1186/1744-9081-3-24 17517138 PMC1891305

[14] McLachlan GJ and Chang SU. Mixture modelling for cluster analysis. Stat Methods Med Res 2004;13, 347–361. 10.1191/0962280204sm372ra 15516030

[15] Chen CC-M, Mengersen KL, Keith JM, Martin NG, and Nyholt DR. Linkage and heritability analysis of migraine symptom groupings: a comparison of three different clustering methods on twin data. Hum Genet 2009;125, 591–604. 10.1007/s00439-009-0652-7 19296132

[16] Greenberg D, Zhang J, Shmulewitz D, Strug L, Zimmerman R, Singh V, et al Construction of the model for the genetic analysis workshop 14 simulated data: genotype-phenotype relationships, gene interaction, linkage, association, disequilibrium, and ascertainment effects for a complex phenotype. BMC Genetics 2005;6, S3 10.1186/1471-2156-6-S1-S3 16451639 PMC1866756

[17] Hoeting JA, Madigan D, Raftery AE, and Volinsky CT. Bayesian model averaging: A tutorial. Stat Sci 1999;14, 382–401.

[18] Kass RE and Raftery AE. Bayes factors. J Am Stat Assoc 1995;90, 773–795. 10.1080/01621459.1995.10476572

[19] Draper D. Assessment and propagation of model uncertainty. J R Stat Soc B 1995;57, 45–97

[20] Dijkstra TK. On Model Uncertainty and its statistical implications. (Springer-Verlag Berlin); 1988.

[21] Chatfield C. Model uncertainty, data mining and statistical inference. J R Stat Soc A 1995;158, 419–466. 10.2307/2983440

[22] Schouwenberg E, Houweling H, Jansen MJW, Kros J, and Mol-Dijkstra JP. Uncertainty propagation in model chains: a case study in nature conservancy. Alterra rapport 001. Alterra, Green World Research, Wageningen; 2000.

[23] Madigan D, Gavrin J, and Raftery AE. Enhancing the predictive performance of Bayesian graphical models. Commun Stat Theory 1995;24, 2271–2292. 10.1080/03610929508831616

[24] Spiegelhalter DJ, Best NG, Carlin BP, and van der Linde A. Bayesian measures of model complexity and fit. J R Stat soc B 2002;64, 583–639. 10.1111/1467-9868.00353

[25] Raftery AE, and Zheng Y. Long-run performance of Bayesian model averaging. Technical Report no. 433, Department of Statistics, University of Washington; 2003.

[26] Raftery AE, Madigan D, and Volinsky CT. Accounting for model uncertainty in survival analysis improves predictive performance. Bayes Stat 1996;5, 323–349.

[27] Raftery AE, Madigan D, and Hoeting JA. Bayesian model averaging for linear regression models. J Am Stat Assoc 1997;92, 179–191. 10.1080/01621459.1997.10473615

[28] Hoeting JA. Methodology for Bayesian model averaging: an update. Proceedings-Manuscripts of Invited Paper Presentations, International Biometric Conference. pp. 231–240; 2002.

[29] Yeung KY, Bumgarner RE, and Raftery AE. Bayesian model averaging: development of an improved multi-class, gene selection and classification tool for microarray data. Bioinformatics 2005;21, 2394–2402. 10.1093/bioinformatics/bti319 15713736

[30] Annest A, Bumgarner R, Raftery A, and Yeung KY. Iterative bayesian model averaging: a method for the application of survival analysis to high-dimensional microarray data. BMC Bioinformatics 2009;10, 17 10.1186/1471-2105-10-72 19245714 PMC2657791

[31] Posada D and Buckley TR. Model selection and model averaging in phylogenetics: advantages of akaike information criterion and Bayesian approaches over likelihood ratio tests. Syst Biol 2004;53, 793 10.1080/10635150490522304 15545256

[32] Fridley Brooke L. Bayesian variable and model selection methods for genetic association studies. Genet Epidemiol 2009;33, 27–37. 10.1002/gepi.20353 18618760

[33] Bernado JM and Smith AFM. Bayesian theory. (New York: Wiley); 1994.

[34] Ott J, Kamatani Y, and Lathrop M. Family-based designs for genome-wide association studies. Nature Rev Genet 2011;12, 465–474. 10.1038/nrg2989 21629274

[35] Godsill SJ. On the relationship between Markov Chain Monte Carlo methods for model uncertainty. J Comput Graph Stat 2001;10, 230–248. 10.1198/10618600152627924

[36] Evans M and Swartz T. Methods for approximating integrals in statistics with special emphasis on Bayesian integration problems. Stat Sci 1995;10, 254–272. 10.1214/ss/1177009938

[37] Gelman A and Meng XL. Simulating normalizing constants: From importance sampling to bridge sampling to path sampling. Stat Sci 1998;13, 163–185. 10.1214/ss/1028905934

[38] Lewis SM and Raftery AE. Estimating Bayes factors via posterior stimulation with the Laplace-Metropolis estimator. J Am Stat Assoc 1997;92, 648 10.2307/2965712

[39] Raftery AE. Hypothesis testing and model selection via posterior simulation In Gilks W, Spiegelhalter DJ, and Richardson S, eds., Practical Markov Chain Monte Carlo (London: Chapman and Hall); 1995.

[40] Marin JM and Robert CP. Bayesian core: a practical approach to computational Bayesian statistics. (Springer Verlag); 2007.

[41] Erosheva EA. Grade of membership and latent structure models with application to disability survey data. Ph.d. Carnegie Mellon University; 2002.

[42] Strehl A and Ghosh J. Cluster ensembles—a knowledge reuse framework for combining multiple partitions. J Mach Learn Res 2003;3, 583–617.

[43] Fern XZ and Brodley CE. Random projection for high dimensional data clustering: A cluster ensemble approach. ICML 2003;3, 186–1937.

[44] Wei Y and McNicholas P. Mixture model averaging for clustering. Advances in Data Analysis and Classification 2015;9(2), 197–217. 10.1007/s11634-014-0182-6

[45] Russell N, Murphy T, and Raftery AE. (under review). Bayesian model averaging in model-based clustering and density estimation. http://arxiv.org/pdf/1506.09035.pdf

[46] Gilks WR, Richardson S and Spiegelhalter DJ. Markov chain Monte Carlo in practice. London: Chapman and Hall; 1996.

[47] Haseman JK and Elston RC. The investigation of linkage between a quantitative trait and a marker locus. Behav Genet 1972;2, 3–19. 10.1007/BF01066731 4157472

[48] Abecasis GR, Cherny SS, Cookson WO, and Cardon LR. Merlin–rapid analysis of dense genetic maps using sparse gene flow trees. Nat. Genet. 2002;30, 97–101. 10.1038/ng786 11731797

[49] Newton MA and Raftery AE. Approximate Bayesian inference with the weighted likelihood bootstrap. J R Stat Soc B 1994;56, 3–48.

[50] Carlin BP and Chib S. Bayesian model choice via Markov chain Monte Carlo methods. J R Stat Soc B 1995;57, 473–484.

[51] Gelfand A. Gibbs sampling. In Encyclopedia of the Statistical Science 1; 1997.

[52] Lord FM. Applications of item response theory to practical testing problems. Routledge; 1980.

[53] Thomas DC, Haile RW, and Duggan D. Recent developments in Genomewide association scans: a workshop summary and review. Am J Hum Genet 2005;77, 337–345. 10.1086/432962 16080110 PMC1226200

