# Supplementary material for: Accurate phenotyping: Reconciling approaches through Bayesian model averaging
Source: PLoS One. 2017 Apr 19;12(4):e0176136. doi: 10.1371/journal.pone.0176136 (PMC5396931; doi:10.1371/journal.pone.0176136)
Supplement: S1 File — (PDF) [file pone.0176136.s001.pdf]

Posterior and conditional distribution of LCA and GoM

Chen et al.

January 12, 2017

Bayesian Latent Class Analysis

The Likelihood of LCA is

$$p(y|p,\lambda) = \prod_i \sum_k p_k \prod_j \lambda_{kj}^{y_{ij}} (1-\lambda_{kj})^{1-y_{ij}}. \tag{1}$$

Let

$$f(Y_i|\lambda_k) = \prod_j \lambda_{kj}^{y_{ij}} (1-\lambda_{kj})^{1-y_{ij}}$$

so that we can write Equation 1 as

$$p(y|p,\lambda) = \prod_i \sum_k p_k f(Y_i|\lambda_k). \tag{2}$$

In this study, we used the following priors for λ and p:

$$\begin{aligned} p &\sim \text{Dirichlet}(\alpha_1, \ldots, \alpha_k) \\ \lambda &\sim \text{Beta}(\alpha, \beta) \end{aligned}$$

To simplify the calculation, we introduced latent variables z_{ik}, each of which takes the value 1 if individual i is assigned to cluster k, and 0 otherwise. Define a joint probability for y and z given p and λ as follows:

$$p(y,z|p,\lambda) = \prod_i \prod_k (p_k \prod_j \lambda_{kj}^{y_{ij}} (1-\lambda_{kj})^{1-y_{ij}})^{z_{ik}}.$$

Note that if we sum over z, we recover Equation1. Using Bayes theorem, the posterior distribution for LCA is proportional to:

$$\begin{aligned} p(y,z|p,\lambda)p(p)p(\lambda) &= p(p)p(\lambda) \prod_i \prod_k (p_k f(Y_i|\lambda_k))^{z_{ik}} \\ &\propto \prod_k p_k^{\alpha_k - 1} \prod_j \lambda_{kj}^{\alpha-1} (1-\lambda_{kj})^{\beta-1} \prod_i (p_k \lambda_{kj}^{y_{ij}} (1-\lambda_{kj})^{1-y_{ij}})^{z_{ik}} \end{aligned}$$

The conditional distributions for model parameters are therefore

$$p(p|y,z,\lambda) \sim \text{Dirichlet}(\sum_i z_{i1} + 1, \ldots \sum_i z_{iK} + 1)$$
$$p(\lambda_{kj}|y,z,p) \sim \text{Beta}(\sum_i y_{ij} z_{ik} + 1, \sum_i z_{ik}(1 - y_{ij}) + 1)$$
$$p(z|y,p,\lambda) \sim \text{Multinomial}(\delta_{i1}, \ldots, \delta_{iK})$$
$$\delta_{ik} = \frac{p_k \prod_j \lambda_{kj}^{y_{ij}} (1 - \lambda_{kj})^{1-y_{ij}}}{\sum_l p_l \prod_j \lambda_{lj}^{y_{ij}} (1 - \lambda_{lj})^{1-y_{ij}}}$$

The derivation of the conditional distributions for GoM can be found in: Erosheva, E. A. (2003). Grade of membership and latent structure models with application to disability survey data. In J.M. Bernardo, M.J. Bayarri, J.O. Berger, A.P. Dawid, D. Heckerman, A.F.M. Smith and M. West (Eds.), *Bayesian Statistics 7*. Oxford: Oxford University Press.
